# Inflammatory bowel disease patients’ perspectives of non-medical needs

**DOI:** 10.1186/s12876-024-03214-x

**Published:** 2024-04-13

**Authors:** Narges Norouzkhani, Mahbobeh Faramarzi, Ali Bahari, Javad Shokri Shirvani, Saeid Eslami, Hamed Tabesh

**Affiliations:** 1 Department of Medical Informatics, Faculty of Medicine, Mashhad University of Medical Sciences, Mashhad, 13944-91388 Iran; 2 Population, Family and Spiritual Health Research Center, Health Research Institute, Babol University of Medical Sciences, Babol, Iran; 3 Department of Internal Medicine, Faculty of Medicine, Mashhad University of Medical Sciences, Mashhad, 13944- 91388 Iran; 4 Department of Internal Medicine, Babol University of Medical Sciences, Babol, 47176-47754 Iran

**Keywords:** Inflammatory bowel diseases, Needs assessment, Exploratory factor analysis, Confirmatory factor analysis

## Abstract

**Background:**

Inflammatory bowel disease (IBD) imposes a huge burden on the healthcare systems and greatly declines the patient’s quality of life. However, there is a paucity of detailed data regarding information and supportive needs as well as sources and methods of obtaining information to control different aspects of the disease from the perspectives of the patients themselves. This study aimed to establish the IBD patients’ preferences of informational and supportive needs through Exploratory Factor Analysis (EFA) and Confirmatory Factor Analysis (CFA).

**Methods:**

IBD patients were recruited from different centers. Considering inclusion and exclusion criteria, 521 participants were filled a predefined questionnaire. This questionnaire was prepared through literature review of the recent well-known guidelines on the needs of IBD patients, which was further approved by the experts of IBD area in three rounds of Delphi consensus. It includes 56 items in four sections of informational needs (25), supportive needs (15), sources of information (7), and methods of obtaining information (9).

**Results:**

In particular, EFA was used to apply data reduction and structure detection. Given that this study tries to identify patterns, structures as well as inter-relationships and classification of the variables, EFA was utilized to simplify presentation of the variables in a way that large amounts of observations transform into fewer ones. Accordingly, the EFA identified five factors out of 25 items in the information needs section, three factors out of 15 items in the supportive needs section, two factors out of 7 items in the information sources section, and two factors out of 9 items in the information presentation methods. Through the CFA, all 4 models were supported by Root Mean Squared Error of Approximation (RMSEA); Incremental Fit Index (IFI); Comparative Fit Index (CFI); Tucker-Lewis Index (TLI); and SRMR. These values were within acceptable ranges, indicating that the twelve factors achieved from EFA were validated.

**Conclusions:**

This study introduced a reliable 12-factor model as an efficient tool to comprehensively identify preferences of IBD patients in informational and supportive needs along with sources and methods of obtaining information. An in-depth understanding of the needs of IBD patients facilitates informing and supporting health service provision. It also assists patients in a fundamental way to improve adaptation and increase the quality of life. We suggest that health care providers consider the use of this tool in clinical settings in order to precisely assess its efficacy.

**Supplementary Information:**

The online version contains supplementary material available at 10.1186/s12876-024-03214-x.

## Introduction

Inflammatory bowel disease (IBD) is known as one of the major chronic and recurrent intestinal disorder that is manifested in two main forms of ulcerative colitis (UC) and Crohn’s disease (CD). They are followed by a wide variety of complications in developed countries and its incidence rate is rising in developing regions [1, 2]. UC causes superficial mucosal inflammation in the colon that leads to ulcerations, toxic mega colon, profuse bleeding, and acute severe colitis (ASC). On the other hand, CD affects all parts of the digestive tract often discontinuously, and is characterized by transmural inflammation, which results in certain problems like abscesses, fibrotic strictures, and fistulas [3]. North America, Europe, and Asian industrialized countries have a higher prevalence rate of UC [4]. Moreover, people over 30 years old are at higher risk of developing UC [5]. In contrast, nearly one-fourth of CD diagnosis occurs during adolescence [6].

Patients with IBD suffer from a wide range of symptoms such as abdominal pain, fatigue, weight loss, diarrhea, and bloody stools or rectorrhagia [7, 8]. The vastness of such kind of problems causes psychological and social impairments that severely disrupts the patient’s normal life [9]. Subsequently, patients experience reduced quality of life because of low self-esteem, poor body image, difficulty in intimate relationships, and decreased productivity [10]. In such circumstances, it is necessary that patients and their caregivers receive adequate and appropriate information and training to deal with the disease and control its sequels [9]. Furthermore, IBD patients are usually on long-term use of medications and invasive interventions. This condition exacerbates the need for extra support and information [11].

Previous studies have shown that the majority of IBD patients prefer to receive their required information through gastroenterologists and the Internet [12, 13]. However, half of the patients have perceived deficiencies in the received information, and thereby, look for more reliable sources [14]. Also, limited data is currently available regarding preferences of IBD patients for different types of needs. Therefore, the present study aimed to establish the preferences of IBD patients for informational and supportive needs besides sources of obtaining information via Confirmatory Factor Analysis (CFA) and Exploratory Factor Analysis (EFA).

## Methods

### Participants and sampling

IBD patients were recruited through different ways. A phone call was made with those patients whose information was recorded in national registries. Also, volunteer patients were participated via announcements in social media. IBD patients in their periodic visits at defined Gastroenterology and Hepatology clinics in different cities (Mashhad, Babol, Amol, Tehran, and Shiraz) were invited too. Inclusion criteria were as the follows: IBD must has been diagnosed in the participants according to the international guideline at least six months prior to the onset of the study, age of ≥ 18 years, ability to communicate in native language with the study team members, and providing written informed consent. Those who were not adhere to the study requirements or unwillingness to keep participation were excluded. Out of 644 included patients, 521 individuals remained in the study. They filled the questionnaire via either paper or online form. Data was recorded in a web-based platform.

Based on the questionnaire’s item count (1:10) and the 15% non-participation rate of patients, the sample size was calculated. A total of 521 patients completed the study instrument, representing a response rate of 80.90%. According to the study of Fincham, “A response rate of approximately 60% should be the goal of researchers for most research“ [15]. Therefore, the response rate is acceptable in quantitative research.

### Ethical consideration

This study was started after obtaining ethical approval from the university and participation was voluntary. Finally, the compiled questionnaire was distributed among qualified patients after the approval of the specialized ethics committee in biomedical research of Mashhad University of medical sciences (IR.MUMS.REC.1400.230) and after obtaining written informed consent.

### Data collection

Data were collected by a structured questionnaire between May 2022 and September 2022. The primary items of this questionnaire items, based on scoping review study [16, 17], guidelines of the American Gastroenterological Association, American College of Gastroenterology, Crohn’s & Colitis Foundation, European Crohn’s and Colitis Organization as well as British Society of Gastroenterology consensus guidelines, on our previous work were scrutinized. A list containing vital needs in informational (56 items) and supportive (36 items) needs besides information sources (19 items) and methods of obtaining information (17 items) was prepared. These 128 items were transformed into corresponding questions and were subjected to three rounds of Delphi consensus to have the experts’ opinions in this regard [18]. In this way, 75 items including 37 information needs, 20 supportive needs, 9 sources of information, and 9 methods of obtaining information were found fundamental by the experts. On this basis, the new questionnaire, which became shortened after checking validity and reliability, was delivered to the IBD patients to acquire their opinions in different sections. We inevitably decided to work on this version in order to not lose any important item. The instrument containing 13 demographic and disease-related questions (sex; age; marital status; ethnicity; education level; employment status; type of disease; duration of disease; patient’s age at the time of diagnosis; current disease status; factors affecting the incidence, recurrence, or exacerbation of the disease from the patient’s point of view; the history of IBD in the family; and the history of GI surgery) and three other sections. These three sections include 56 items and 4 open questions as follows (Supplement Tables 1, 2, 3 and 4): information needs of patients with IBD (25 questions), supportive needs of patients with IBD (15 questions), and information sources and information presentation methods on patients with IBD (16 questions). The questions are ranked on a 5-point Likert scale (0-no need and 4-strong need). This questionnaire also evaluates the acceptance of patients from the information sources and presentation of Information methods to patients with IBD using a 5-point scale (0-non-acceptance of the source and 4-full acceptance of the source). Fifteen experts in the fields of gastrointestinal and liver diseases in adults, psychiatrists or clinical psychologists, and members of the nursing and health information technology faculty reviewed the items to confirm the content validity of the research instrument and to ensure that the purpose of the study was consistent with it.

The process was governed by Davis’s four suggested processes for the instrument development for this study: [1] concept identification using literature; [2] item design by deciding on the readability, blueprint, item authoring, format, and scoring; [3] validity of the tool by expert review; factor analysis (CFA and EFA); and [4] instrument reliability [19].

### Data analysis

Data analysis was performed using SPSS V26 for EFA, and Amos V26 was applied for the CFA. Design requirements of the study and preferred statistical analyses were founded on the basis of COSMIN checklist [20] and we used the STROBE cross sectional checklist when writing our report [21]. We followed the methods of Alexis Harerimana et al. 2020 [22] for data analyses. Factor analysis (FA) is one of the most extremely useful methods to psychometrically test instruments in methodological studies, which is conducted via two methods: EFA and CFA [23, 24].In the present study, EFA and CFA were applied to construct validity and identify the preferences of patients with IBD for their important needs. Specifically, EFA was performed as a pre-test to assess the construct validity of the items within the questionnaire as well as reducing its dimension. At the first stage, the standard normal distribution was assessed by verification of the kurtosis (-7 to + 7) and skewness (‐2 to + 2) that should be within the acceptable range [25]. In the primary EFA phase, data screening was done using Bartlett’s sphericity test (0.05) and Kaiser-Meyer-Olkin (KMO) measure of sampling adequacy (> 0.5) [26]. The parallel analysis (PA) in terms of Principal Component Analysis (PCA) was carried out to indicate the number of factors that is maintained in the model [27]. Fifty-six items with a 15% non-participation rate and a sample size of 644 were considered. A total of 644 questionnaires were delivered to the IBD patients. Among them, 616 questionnaires were filled. Number of questionnaires that was omitted due to lack of cooperation and/or incompleteness was 95 resulting to a net 521 complete questionnaires (response rate = 80.90%).

Also, factors with Eigenvalue of more than one were examined. EFA was proceeded relying on the 12 identified factors [27]. The CFA method was conducted to validate the factors associated with the information needs, supportive needs, information sources, and information presentation methods, and some indices were applied to indicate the extent of the model fitness.

The conditions for the best fitness was selected from the relevant indices [28]. In our research, the goodness of model was assessed using indices such as comparative fit index (CFI ≥ 0.90); Chi-square/Degree of Freedom (CMIN/DF ≤ 5); Turker-Lewis Index (TLI ≥ 0.90); the incremental index of fit (IFI, > 0.090); Standardized Root Mean Square Residual (SRMR ≤ 0.08), Root Mean Squared Error of Approximation (RMSEA, ≤ 0.08), and Goodness of Fit Index (GFI ≥ 0.90). Based on EFA results, all remained variables in EFA models were regarded in generating CFA models. It should be noted that some changes were implemented based on the fitness indices, and factor loading for each item was also examined. CFA and EFA were performed on the same data [29, 30].

## Results

### Biographical details of participants

As shown in Supplement Table 5, a total of 521 patients with IBD participated in this study with a mean age of 37.57 years (SD = 11.54) and 69.87% were female. Also, 66.99% of them were married. The average duration of their disease was 8.99 (SD = 7.04) years. Among the participants, 67.37% had UC, and 59.69% had inactive disease. Also, 62.19% had no history of IBD in the family and 85.60% had no history of GI surgery.

### Descriptive statistics of the items

For the whole questions within the questionnaire, Cronbach’s alpha and McDonald’s Omega were respectively 0.928 and 0.917. While the former was higher than the minimum acceptable reliability of 0.70 [31] and shows high reliability of the instrument, the latter indeed reinforces high reliability between variables. Specifically, McDonald’s Omega was calculated for each section of informational needs (0.928), supportive needs (0.92), sources of information (0.88), and methods of obtaining information (0.862). Similarly, Interclass Correlation Coefficient was calculated through Two-way random approach for the whole questionnaire (0.928) as well as for the informational Sect. (0.931), supportive Sect. (0.920), sources of information (0.888), and methods of obtaining information (0.862). Fifty-six items were assessed using the skewness, kurtosis, mean, and standard deviation. The total mean was 3.99 (S.D = 1.05), ranging from 3.69 to 4.33. The skewness (< 2) and ranged from − 1.23 to -0.31, and the kurtosis (< 7) ranged from − 1.19 to 1.33 (Supplement Table 6).

### Exploratory factor analysis (EFA)

EFA was done through the data obtained from the 521 cases using the PCA, the Rotation Method being Oblimin with Kaiser Normalization. The KMO index of 0.60 is considered acceptable for factor analysis [24, 32]. In this study, as shown in Table 1, in all 4 indicators, the value of KMO was greater than 0.6 and the results of Bartlett’s test of Sphericity were significant for all 4 indicators. Therefore, the sample size of the study was appropriate and the implementation of exploratory factor analysis was allowed, suggesting a powerful relationship among the variables and the appropriateness of data to conduct an EFA. Using PA, twelve factors with Eigenvalues > 1 were obtained, as suggested by Horn [33], which is a recommended method to assess the number of factors. The twelve factors were obtained as follows:

**Table 1:** KMO and Bartlett’s test

	Kaiser-Meyer-Olkin Measure of Sampling Adequacy (KMO)	Bartlett’s test of sphericity		
		Approx. Chi-square	Df	P-value
Information needs	0.935	7613.147	300	< 0.001
Information sources	0.830	2284.006	21	< 0.001
Information presentation methods	0.878	1736.258	36	< 0.001
Supportive needs	0.914	4961.661	105	< 0.001

#### Information needs

As shown in Table 2, based on the eigenvalues, five factors were extracted in this section. These five factors explained 66.44% of the total variance. The five factors were named as follows: Factor 1:Self-management information (A1) with eight items with a factor loading between 0.764 and 0.837. Factor 2: Preventive and supportive care information (A2) with six items and factor loading between 0.667 and 0.781. Factor 3: Life-style and risky behaviors information (A3) with five items and factor loading between 0.604 and 0.752. Factor 4: Medical information (A4) with four items the factor loading between 0.613 and 0.713.Factor 5: Healthcare provider team information (A5) with two items and the factor loading between 0.821 and 0.836. Cronbach’s alpha ensured the reliability of the factors; factors > 0.70, indicated a good reliability (factor 1 = 0.941; factor 2 = 0.893; factor three = 0.801; factor four = 0.705; factor five = 0.822).

**Table 2:** Factor loadings for information needs

Items	Factors				
	F1	F2	F3	F4	F5
Tip for Psychological factors control	0.837				
Treatment	0.816				
Pain and symptom management	0.810				
Symptoms/Clinical manifestations of IBD	0.806				
Tip for coping	0.799				
Disease management	0.797				
The prevention of relapse action in relapse	0.773				
Risk factors of flares	0.764				
Colorectal cancer		0.781			
Risk of infection		0.767			
Vaccinations in IBD		0.733			
Gynecological issues		0.713			
Extra-intestinal manifestations and IBD complication		0.694			
Covid-19 and IBD		0.667			
Nutritional deficiencies			0.752		
IBD-related travel information			0.678		
Risky behaviors (smoking, alcohol, drug, Tobacco)			0.675		
Exercise and physical activity			0.659		
Nutrition			0.604		
Diagnostic methods				0.713	
IBD evolution and further course				0.674	
Long-term consequences				0.640	
Cause of IBD				0.613	
When connect to IBD team					0.836
Hospitals and Doctors information					0.821

#### Information sources

As shown in Table 3, based on the eigenvalues, two factors were extracted in this section. These two factors explained 75.04% of the total variance. The two factors were named as follows: Factor 1: Scientific resources and support services (B1) with four items with a factor loading between 0.664 and 0.858. Factor 2: Healthcare provider team (B2) with three items and factor loading between 0.752 and 0.899. Cronbach’s alpha ensured the reliability of the factors; factors > 0.70 indicated good reliability (factor one = 0.858; factor 2 = 0.879).

**Table 3:** Factor loadings for information sources

Items	Factors	
	F1	F2
Educational website	0.858	
Scientific researches and articles in scientific and medical journals	0.846	
Hospitals or IBD clinic	0.780	
Counseling and support groups	0.664	
Health professionals team		0.899
Gastroenterologists		0.898
IBD nurse		0.752

#### Information presentation methods

Two factors were extracted in this section. These factors explained 59.28% of the total variance. The two factors were named as follows: Factor 1: educational Media (C1) with four items with a factor loading between 0.674 and 0.799. Factor 2: Social Media and telephone information service (C2) with five items and factor loading between 0.516 and 0.782 (Table 4). Cronbach’s alpha ensured the reliability of the factors; factors > 0.70, indicated a good reliability (factor 1 = 0.799; factor 2 = 0.801).

**Table 4:** Factor loadings for information presentation methods

Items	Factors	
	F1	F2
Brochures or booklet	0.799	
Educational videos	0.791	
Mobile applications	0.720	
TV or radio	0.674	
Websites		0.782
Social medias (telegram, whatsapp)		0.758
Email		0.744
Interactive voice response		0.557
Short messaging service		0.516

#### Supportive needs

Three factors were extracted in this section. These factors explained 70.13% of the total variance. The three factors were named as follows: Factor 1: Patient-Physician Communication needs (D1) with six items with a factor loading between 0.739 and 0.811. Factor 2: Psychosocial needs (D2) with five items and factor loading between 0.756 and 0.818.Factor 3: Facility’s needs (D3) with four items and factor loading between 0.764 and 0.813 (Table 5). Cronbach’s alpha ensured the reliability of the factors; factors > 0.70 indicated good reliability (factor 1 = 0.899; factor 2 = 0.901; factor 3 = 0.871).

**Table 5:** Factor loadings for supportive needs

Items	Factors		
	F1	F2	F3
Easy and immediate access to specialist staff	0.800		
Monitoring and follow-up	0.791		
Shared-decision making	0.767		
Support and patient-physician interaction	0.811		
Multidisciplinary care services	0.758		
Information-sharing coordination between physician and patients	0.739		
Disease management		0.818	
Ability to obtain psychological skills		0.787	
Mental health support		0.767	
Social health support systems		0.771	
Family or Caregivers supports		0.756	
Facilities support			0.775
Intimacy support			0.823
Insurance support			0.764
Patients and Caregivers education			0.813

### Confirmatory factor analysis (CFA)

#### Measurement model

Validation of the identified twelve factors of the IBD patient’s needs CFA was conducted. For cross-validation of the found factors CFA was performed, and multicollinearity was found as independent variables (Five factors for information needs, two for information sources, two for information presentation methods, and two for supportive needs).

**Fig. 1:**
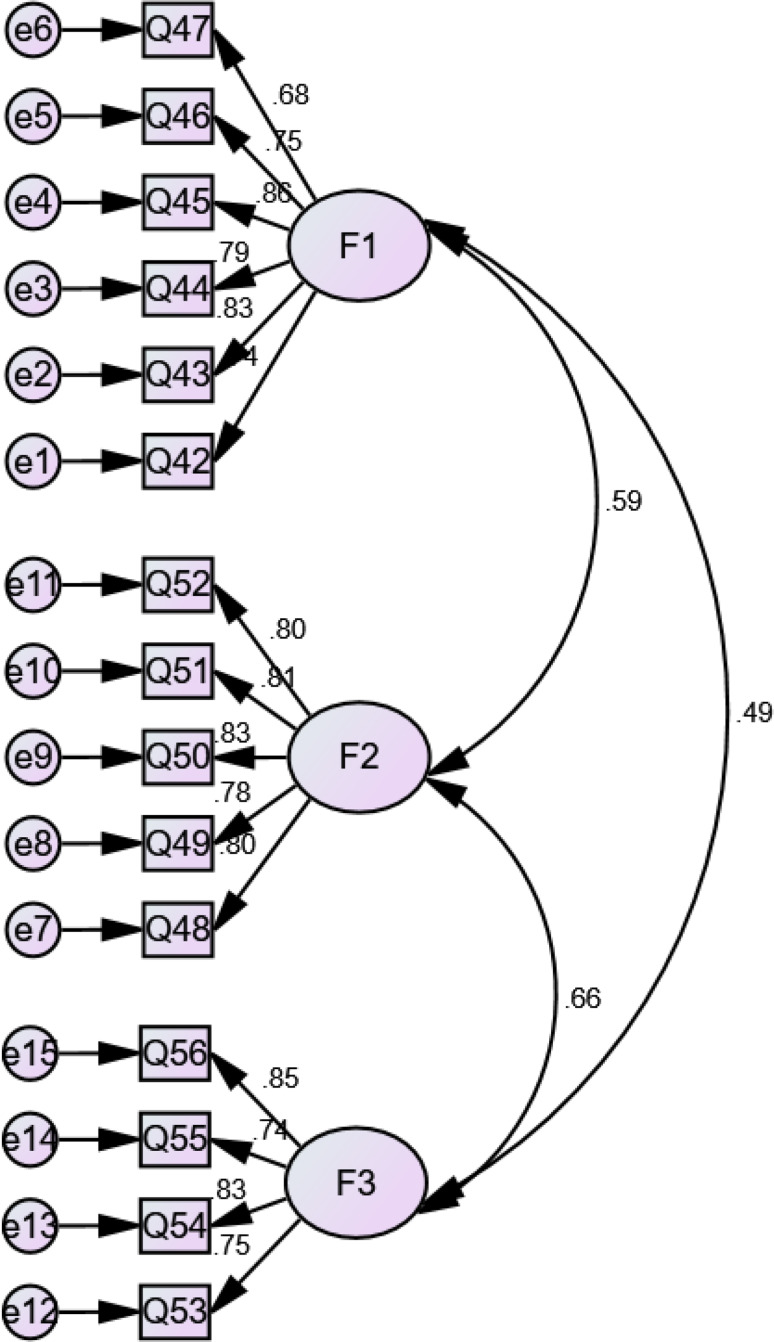
CFA Model for the IBD patients’ preferences for information needs (with standardized estimates). Chi-square goodness of fit ((χ2 / d.f = 1.94)); CFI = 0.972 (> 0.90); IFI = 0.97 (> 0.90); TLI = 0.968 (> 0.90); RMSEA = 0.043 (< 0.080); SRMR = 0.033 (< 0.08)

**Fig. 2:**
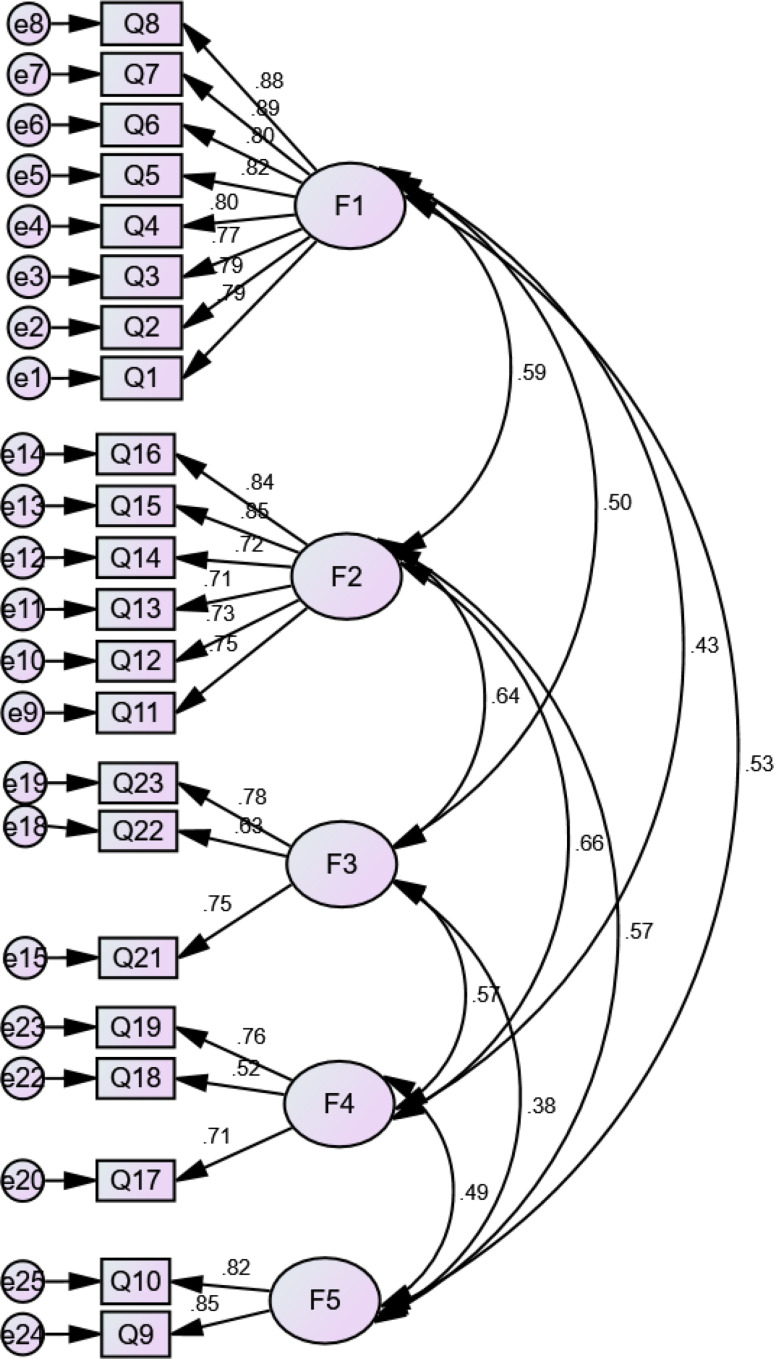
CFA Model for the IBD patients’ preferences information sources needs (with standardized estimates). Chi-square goodness of fit ((χ2 / d.f = 5.1)); CFI = 0.970 (> 0.90); IFI = 0.97 (> 0.90); TLI = 0.943 (> 0.90); RMSEA = 0.087 (< 0.080); SRMR = 0.047 (< 0.08)

**Fig. 3:**
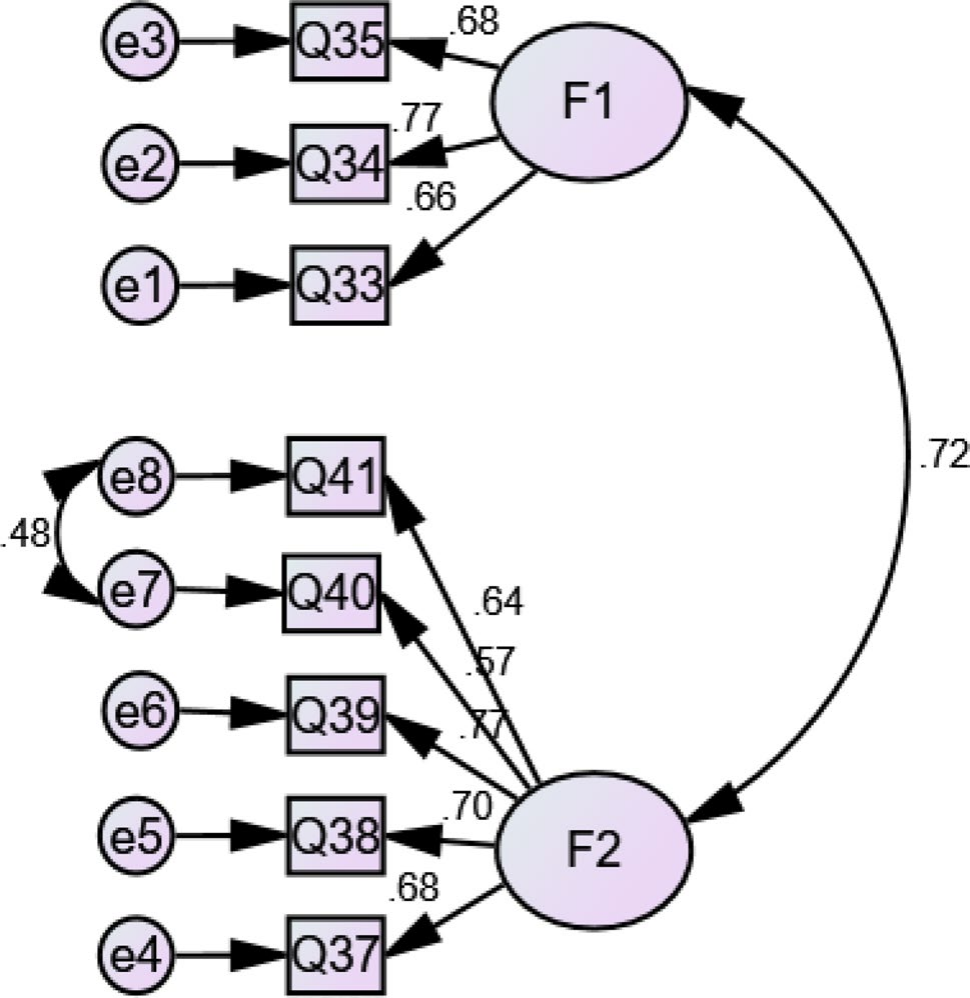
CFA Model for the IBD patients’ preferences for information presentation methods (with standardized estimates). Chi-square goodness of fit ((χ2 / d.f = 2.07)); CFI = 0.987 (> 0.90); IFI = 0.987 (> 0.90); TLI = 0.98 (> 0.90); RMSEA = 0.045 (< 0.080); SRMR = 0.036 (< 0.08)

**Fig. 4:**
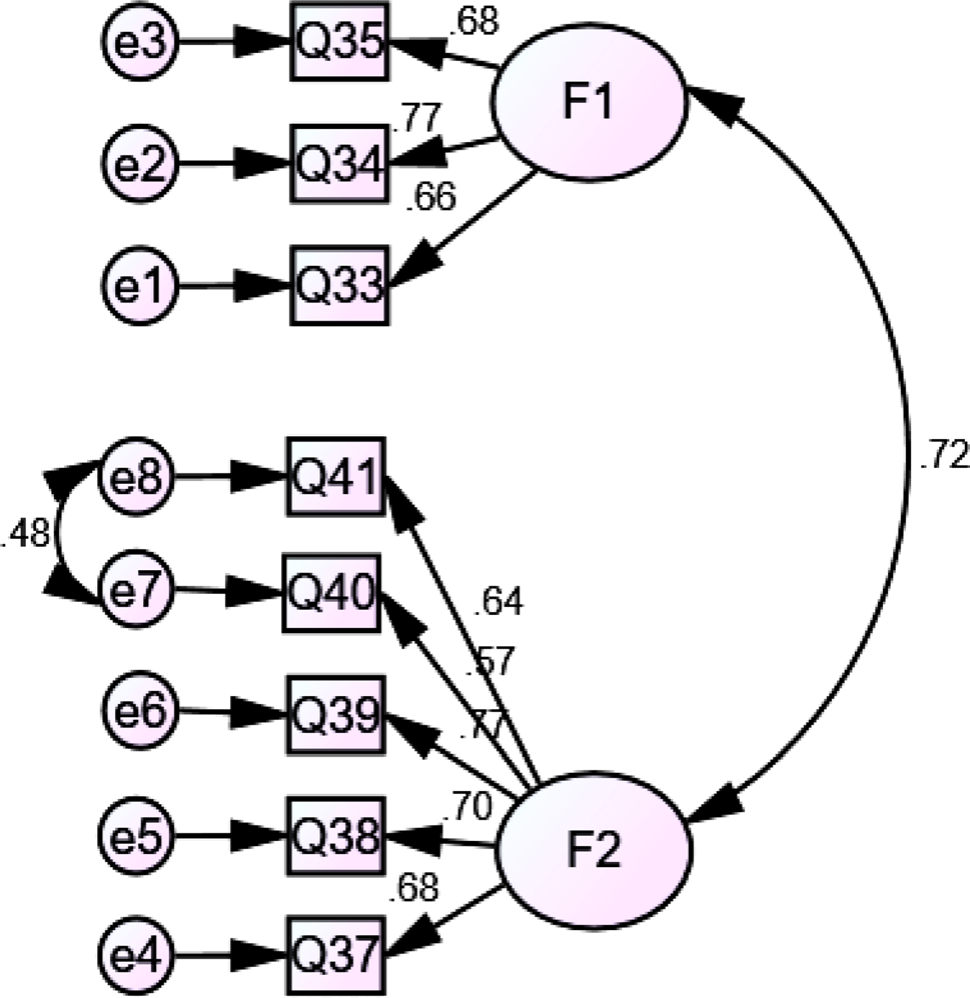
CFA Model for the IBD patients’ preferences for supportive needs (with standardized estimates). Chi-square goodness of fit ((χ2 / d.f = 4.17)); CFI = 0.944 (> 0.90); IFI = 0.94 (> 0.90); TLI = 0.93 (> 0.90); RMSEA = 0.078 (< 0.080); SRMR = 0.046 (< 0.08)

A positive correlation was found between the factors, with estimates being between *r* = 0.147 and *r* = 0.44 for information needs, from *r* = 0.374 for information sources, from *r* = 0.47 for information presentation methods, and *r* = 0.23 to *r* = 0.44 for supportive needs. Moreover, a significant relationship was detected among the factors (*p* < 0.05 and *p* < 0.01), which showed independent variables. Regarding information needs, information sources, information presentation methods, and supportive needs, the results for standardized estimates were from β = 0.522 to β = 0.89, β = 0.68 to β = 0.963, β = 0.568 to β = 0.774, and β = 0.68 to β = 0.857, respectively with *p* < 0.001. The Chi-square goodness of fit test was not significant although the Chi-square test tends to be a statistically significant test, it is highly sensitive against model fit and rejects the model when the model or sample is large [34–36]. Figures 1, 2, 3 and 4 indicate latent variables as well as their relevant observational variables of the final models.

#### Assessment of measurement model: model fit indicators

Based on Figs. 1, 2, 3 and 4, all of the items showed strong factor loadings (above 0.5) with their corresponding latent constructs. Due to the limitations of the Chi-square, other multiple indices including GFI; CFI; IFI; TLI; RMSEA, were used to assess the model fit. Figures 1, 2, 3 and 4 show the values of the fit indices for each factor and are within acceptable ranges for all indices, which indicated that factors obtained from the EFA were validated so that the model efficiency is verified.

## Discussion

IBD, a chronic immune-mediated illness of the gastrointestinal tract, adversely modifies the mental and physical health of the affected patients [37]. The chronic nature of IBD and the complexities in managing the disease make it essential to provide support and necessary information in different required aspects such as treatment options, symptom improvement, safe medication use, and maximum comfort [38–40]. Patients with IBD use different resources and have various preferences to meet such needs [41, 42]. Exploratory and confirmatory analysis determine those important needs and preferences of IBD patients. In the present study, a model was established based on 12 important factors identified by EFA: **five** factors in the information needs section including self-management information (factor 1), preventive and supportive care information (factor 2), lifestyle and risky behavior information (factor 3), medical information (factor 4), and health care provider information (factor 5); **two** factors in the information source section including scientific resources and support service (factor 1) and health care provider team (factor 2); **two** factors in the methods of information’s presentation section including educational media (factor 1), social Media and telephone information service (factor 2); and finally, **three** factors in the supportive needs section including patient-communication needs (factor 1), psychosocial support (factor 2), and facilities supports (factor 3). The aforementioned factors in the model have explained at least 60% of the total variance. Also, the confirmatory factor analyses approved the current model as the general one for different needs of patients with IBD.

Several studies have acknowledged that informational needs are the top priority ones in IBD patients [12, 42, 43]. From the patients’ point of view, information is needed in various areas including, but not limited to, medical, self-management, receiving care (preventive or supportive), and communication with the health care team. Unfortunately, such information is either lacking or not received properly to the patients [41, 44]. To overcome these challenges, health policymakers should take regular measures in order to improve the methods of receiving acceptable information to the patients by implementing innovative ways.

Majority of the patients eagerly seek to establish a consistent patient-physician communication in order to meet their informational needs [12, 41]. Having sufficient amounts of information improves self-management in IBD patients [12]. Easy access to proper information has a positive impact on therapeutic outcomes and quality of life, and is related to emotional adjustment [45–47]. Information can be presented to the patients in various formats via books, brochures, social media, and direct contact with specialists through phone or email [48].

Supportive needs are also of critical importance since they help patients to deal with the illness and its consequences [49]. IBD patients face with different financial, psychological, and social problems, which shows the value of supportive needs. For instance, many patients with IBD have lost their jobs due to disease sequels such as depression and anxiety [50, 51], and supportive help of psychiatrists is effective in reducing the upcoming financial burden imposed to IBD patients [52]. Hence, patients need to receive pertinent support from insurance and health organizations, and psychosocial support from their relatives and healthcare providers.

In one study, patients’ experiences of living with IBD were studied with a focus on information and support needs. Patients were frustrated about prolonged diagnosis process, which becomes exacerbated by misdiagnosis and negative impact on quality of life. Loss of trust between healthcare professionals and increased feelings of fear results from lack of information that causes negative effects on patients’ self-management of the disease. Also, the study highlighted the importance of emotional and practical support from partners and family members. Moreover, support that was provided from nurses and surgeons was considered as the highly valued ones. Patients declared that access to information and support improves their life and helps to regain trust in healthcare practitioners [14].

In a recent study, initial medical unmet needs were explored in UC patients. Out of 18, four needs were attributed to inability to lead a normal life. It was declared that better understanding of patients’ view is essential for handling the impact of UC on the life. A further three of them related to the importance of early diagnostic and therapeutic approaches. Another three needs were about new treatment alternatives in these patients. Seven unmet needs dealt with drawbacks of current treatments. Other remaining needs have focused on education of healthcare practitioners and raising awareness regarding development, publication, and dissemination of scientific research for different treatment options. At the end, the authors stated that there are considerable number of critical problems for management of UC that need to be addressed in future research [53].

In another study, challenges of patients with IBD for living and managing of the disease were investigated. Physical symptoms like pain, frequency of bowel motions, urgency, and diarrhea were identified as the one of the major challenges. Accordingly, subsequent impacts of these challenges on different aspects of patients’ life such as social isolation, psychological fragility, and reduced educational and professional opportunities were noted. Also, findings revealed that patients experienced the absence of meaningful support as a serious life challenge mainly from family and friends, not that from healthcare professionals. However, further researches were needed for elucidation of support interventions from healthcare providers and the effect of such helps on self-management of the challenges [54].

Limitations of this study should be acknowledged too. One of the inherent limitations of such studies, self-report assessment, is reporting bias. However, high response rate attenuates this weakness and promotes this notion that the sample population is a good representative of the overall IBD patients. Furthermore, only IBD patients who agreed to fill the questionnaire were included, and this may cause selection bias. Generalizability of the findings may be restrained by unique characteristics of the population. Needs, preferences, and beliefs of one population are not similar to other peers from other countries, societies, and cultures. As the questionnaire of the study was filled through online system other than manually, some difficulties and errors may be occurred for the users in terms of fidelity and careful responding to the items.

Needs and preferences of IBD patients that have been identified in this study should be evaluated with regard to effectiveness of information resources and presentation methods. Future investigations should be focused on how factor structure of the needs and preferences identified in the current study can be extended to other populations. Needs and preferences of IBD patients in younger age or under the legal age of 18 could be also the subject of future studies.

## Conclusions

This study generated a model based on 12 factors for measuring the needs of IBD patients in four subscales of information and supportive needs besides sources and methods of providing information. The findings can be applied for preparing the healthcare professional teams to properly meet the needs and efficiently decrease the psychological burden on the patients and their caregivers.

### Electronic supplementary material

Below is the link to the electronic supplementary material.

Supplementary Material 1

## Data Availability

The original data presented in the study are included in the article. The data that support the findings of this study are available from [Narges Norouzkhani] but restrictions apply to the availability of these data, which were used under license for the current study, and so are not publicly available. Data are however available from the authors upon reasonable request and with permission of [Hamed Tabesh and Narges Norouzkhani].

## Declarations

All participants provided Informed consent for the study.
