# Supplementary material for: Inflammatory bowel disease patients’ perspectives of non-medical needs
Source: BMC Gastroenterol. 2024 Apr 13;24:134. doi: 10.1186/s12876-024-03214-x (PMC11016217; doi:10.1186/s12876-024-03214-x)
Supplement: Supplementary file 1 — Supplementary Material 1 [file 12876_2024_3214_MOESM1_ESM.docx]

**Supplementary materials**

**Suppl Table 1.** Information needs questionnaire of patients with inflammatory bowel disease.

| In your opinion, how important are these information in educating and meeting the information needs of patients with inflammatory bowel disease? | | | | | | |
| --- | --- | --- | --- | --- | --- | --- |
|  |  | Preference range | | | | |
|  |  | Very low  (1) | Low  (2) | Moderate  (3) | High  (4) | Very high  (5) |
| 1 | Cause of IBD |  |  |  |  |  |
| 2 | IBD evolution and further course |  |  |  |  |  |
| 3 | Long-term consequences |  |  |  |  |  |
| 4 | Symptoms/Clinical manifestations of IBD |  |  |  |  |  |
| 5 | Risk factors of flares |  |  |  |  |  |
| 6 | Risky behaviors (smoking,alcohol,drug,Tobacco) |  |  |  |  |  |
| 7 | The prevention of relapse action in relapse |  |  |  |  |  |
| 8 | Pain and symptom management |  |  |  |  |  |
| 9 | Disease management |  |  |  |  |  |
| 10 | Tip for Coping and living inflammatory bowel disease |  |  |  |  |  |
| 11 | Tip for Psychological factors control |  |  |  |  |  |
| 12 | Extra-intestinal manifestations and IBD complication |  |  |  |  |  |
| 13 | Colorectal cancer |  |  |  |  |  |
| 14 | Infection risk |  |  |  |  |  |
| 15 | Treatment |  |  |  |  |  |
| 16 | Nutritional deficiencies |  |  |  |  |  |
| 17 | Nutrition |  |  |  |  |  |
| 18 | Exercise and physical activity |  |  |  |  |  |
| 19 | IBD-related travel information |  |  |  |  |  |
| 20 | Diagnostic methods including (types of diagnostic methods, risk of diagnostic methods and information on the interpretation of diagnostic tests) |  |  |  |  |  |
| 21 | Covid-19 and IBD |  |  |  |  |  |
| 22 | Vaccinations in IBD |  |  |  |  |  |
| 23 | Hospitals and Doctors information |  |  |  |  |  |
| 24 | when connect to IBD team |  |  |  |  |  |
| 25 | Gynecological issues |  |  |  |  |  |

**Suppl Table 2.** Supportive needs questionnaire of patients with inflammatory bowel disease.

|  | In your opinion, how preference are these items in supporting patients with inflammatory bowel disease? | | | | | |
| --- | --- | --- | --- | --- | --- | --- |
|  |  | Preference range | | | | |
|  |  | Very low  (1) | Low  (2) | Moderate  (3) | High  (4) | Very high  (5) |
| 1 | Mental health support |  |  |  |  |  |
| 2 | Ability to obtain psychological skills |  |  |  |  |  |
| 3 | Disease management |  |  |  |  |  |
| 4 | Patients and Caregivers education |  |  |  |  |  |
| 5 | Social health support systems |  |  |  |  |  |
| 6 | Family or Caregivers supports |  |  |  |  |  |
| 7 | Intimacy support |  |  |  |  |  |
| 8 | Facilities support |  |  |  |  |  |
| 9 | Insurance support |  |  |  |  |  |
| 10 | Multidisciplinary care services |  |  |  |  |  |
| 11 | Information-sharing coordination between physician and patients |  |  |  |  |  |
| 12 | Shared-decision making |  |  |  |  |  |
| 13 | Support and patient-physician interaction |  |  |  |  |  |
| 14 | Easy and immediate access to specialist staff |  |  |  |  |  |
| 15 | Monitoring and follow-up |  |  |  |  |  |

**Suppl Table 3.** Information sources needs questionnaire of patients with inflammatory bowel disease.

| In your opinion, how important these sources of obtaining information useful for patients with inflammatory bowel disease? | | | | | | |
| --- | --- | --- | --- | --- | --- | --- |
|  |  | Preference range | | | | |
|  |  | Very low  (1) | Low  (2) | Moderate  (3) | High  (4) | Very high  (5) |
| 1 | Health professionals team |  |  |  |  |  |
| 2 | Gastroenterologists |  |  |  |  |  |
| 3 | IBD nurse |  |  |  |  |  |
| 4 | Hospitals or IBD clinic |  |  |  |  |  |
| 5 | Educational website |  |  |  |  |  |
| 6 | Counseling and support groups |  |  |  |  |  |
| 7 | Scientific researches and articles in scientific and medical journals |  |  |  |  |  |

**Suppl Table 4.** Methods needs questionnaire of patients with inflammatory bowel disease.

| In your opinion, how important these presentation methods for patients with inflammatory bowel disease? | | | | | | |
| --- | --- | --- | --- | --- | --- | --- |
|  |  | Preference range | | | | |
|  |  | Very low  (1) | Low  (2) | Moderate  (3) | High  (4) | Very high  (5) |
| 1 | Brochures or booklet |  |  |  |  |  |
| 2 | TV or radio |  |  |  |  |  |
| 3 | Educational videos |  |  |  |  |  |
| 4 | Websites |  |  |  |  |  |
| 5 | Social medias (telegram, WhatsApp) |  |  |  |  |  |
| 6 | Email |  |  |  |  |  |
| 7 | Mobile applications |  |  |  |  |  |
| 8 | Short messaging service |  |  |  |  |  |
| 9 | Interactive voice response |  |  |  |  |  |

**Suppl Table 5.** Patient characteristics (N=521).

|  | Total (N=521) |
| --- | --- |
|  |  |
| **Age** | 37.57 (SD=11.54) |
| **Sex** |  |
| Male | 157 (30.13) |
| Female | 364 (69.87) |
| **Marital status** |  |
| Single | 168 (32.24) |
| Married | 349 (66.99) |
| Other | 4 (0.77) |
| **Type of Disease** |  |
| Ulcerative Colitis (UC) | 351 (67.37) |
| Crohn's disease (CD) | 170 (32.63) |
| **Duration of disease (year)** | 8.99 (SD=7.04) |
| <5 | 169 (32.44) |
| 5-10 | 188 (36.08) |
| 11-15 | 85 (16.31) |
| 16-20 | 30 (5.77) |
| >=20 | 49 (9.40) |
| **The current state of the disease** |  |
| Active | 210 (40.31) |
| Inactive | 311 (59.69) |
| **History of IBD in the family** |  |
| Yes | 197 (37.81) |
| No | 324 (62.19) |
| **History of GI surgery** |  |
| Yes | 75 (14.4) |
| No | 446 (85.60) |

**IBD:** Inflammatory bowel disease, **GI:** Gastrointestinal

Values are given as a number (percentage) for categorical variables.

**Suppl Table 6.** Descriptive statistics (mean and standards deviation) (n = 521).

| Items' codes | Mean | Std. Deviation | Skewness | | Kurtosis | |
| --- | --- | --- | --- | --- | --- | --- |
|  | Statistic | Statistic | Statistic | Std. Error | Statistic | Std. Error |
| VAR00001 | 3.9271 | 1.05724 | -0.648 | 0.107 | -0.542 | 0.214 |
| VAR00002 | 3.952 | 1.05957 | -0.81 | 0.107 | -0.145 | 0.214 |
| VAR00003 | 3.8714 | 1.09403 | -0.716 | 0.107 | -0.368 | 0.214 |
| VAR00004 | 3.9789 | 1.08554 | -0.854 | 0.107 | -0.16 | 0.214 |
| VAR00005 | 4.0672 | 1.05489 | -0.934 | 0.107 | -0.061 | 0.214 |
| VAR00006 | 3.9539 | 1.07854 | -0.776 | 0.107 | -0.324 | 0.214 |
| VAR00007 | 4.0979 | 1.03776 | -0.964 | 0.107 | 0.014 | 0.214 |
| VAR00008 | 4.0557 | 1.03532 | -0.842 | 0.107 | -0.28 | 0.214 |
| VAR00009 | 4.2111 | 0.96753 | -1.021 | 0.107 | 0.214 | 0.214 |
| VAR00010 | 4.1152 | 0.92309 | -0.834 | 0.107 | 0.153 | 0.214 |
| VAR00011 | 4.1478 | 1.02246 | -1.166 | 0.107 | 0.701 | 0.214 |
| VAR00012 | 4.0365 | 0.96507 | -0.962 | 0.107 | 0.55 | 0.214 |
| VAR00013 | 3.9424 | 1.02307 | -0.89 | 0.107 | 0.309 | 0.214 |
| VAR00014 | 3.9559 | 0.99806 | -0.878 | 0.107 | 0.302 | 0.214 |
| VAR00015 | 4.1094 | 0.91857 | -0.921 | 0.107 | 0.358 | 0.214 |
| VAR00016 | 4.1555 | 0.90978 | -0.881 | 0.107 | 0.066 | 0.214 |
| VAR00017 | 4.023 | 0.99781 | -0.967 | 0.107 | 0.465 | 0.214 |
| VAR00018 | 3.9117 | 1.10239 | -0.819 | 0.107 | -0.159 | 0.214 |
| VAR00019 | 3.9635 | 1.01176 | -0.9 | 0.107 | 0.306 | 0.214 |
| VAR00020 | 3.7198 | 1.18085 | -0.667 | 0.107 | -0.473 | 0.214 |
| VAR00021 | 3.9655 | 1.07362 | -0.961 | 0.107 | 0.366 | 0.214 |
| VAR00022 | 3.8369 | 1.1512 | -0.832 | 0.107 | -0.072 | 0.214 |
| VAR00023 | 4.048 | 1.02451 | -0.98 | 0.107 | 0.409 | 0.214 |
| VAR00024 | 3.8464 | 0.88548 | -0.913 | 0.107 | 1.325 | 0.214 |
| VAR00025 | 4.2265 | 0.952 | -1.231 | 0.107 | 1.128 | 0.214 |
| VAR00026 | 3.8906 | 0.99302 | -0.501 | 0.107 | -0.763 | 0.214 |
| VAR00027 | 4.1094 | 0.99302 | -0.812 | 0.107 | -0.3 | 0.214 |
| VAR00028 | 3.9309 | 0.98597 | -0.622 | 0.107 | -0.621 | 0.214 |
| VAR00029 | 3.9655 | 1.01278 | -0.745 | 0.107 | -0.387 | 0.214 |
| VAR00030 | 3.7294 | 1.14088 | -0.313 | 0.107 | -1.187 | 0.214 |
| VAR00031 | 3.7179 | 1.16975 | -0.522 | 0.107 | -0.755 | 0.214 |
| VAR00032 | 3.8292 | 1.14337 | -0.623 | 0.107 | -0.661 | 0.214 |
| VAR00033 | 4.0154 | 1.04686 | -0.737 | 0.107 | -0.536 | 0.214 |
| VAR00034 | 4.1305 | 1.00108 | -0.991 | 0.107 | 0.17 | 0.214 |
| VAR00035 | 4.0096 | 1.04048 | -0.873 | 0.107 | 0.04 | 0.214 |
| VAR00036 | 4.142 | 1.02045 | -0.952 | 0.107 | -0.211 | 0.214 |
| VAR00037 | 4.2534 | 1.02334 | -1.215 | 0.107 | 0.42 | 0.214 |
| VAR00038 | 4.0557 | 1.05191 | -0.858 | 0.107 | -0.2 | 0.214 |
| VAR00039 | 4.261 | 0.98693 | -1.144 | 0.107 | 0.263 | 0.214 |
| VAR00040 | 4.2226 | 0.9529 | -1.007 | 0.107 | 0.045 | 0.214 |
| VAR00041 | 4.3321 | 0.92565 | -1.217 | 0.107 | 0.454 | 0.214 |
| VAR00042 | 3.9136 | 1.1069 | -0.563 | 0.107 | -0.783 | 0.214 |
| VAR00043 | 3.9367 | 1.13418 | -0.685 | 0.107 | -0.652 | 0.214 |
| VAR00044 | 3.7985 | 1.24795 | -0.621 | 0.107 | -0.806 | 0.214 |
| VAR00045 | 4.048 | 1.09527 | -0.809 | 0.107 | -0.5 | 0.214 |
| VAR00046 | 4.1075 | 1.07236 | -0.985 | 0.107 | 0.079 | 0.214 |
| VAR00047 | 3.9655 | 1.11232 | -0.799 | 0.107 | -0.216 | 0.214 |
| VAR00048 | 4.0288 | 0.98505 | -0.773 | 0.107 | -0.078 | 0.214 |
| VAR00049 | 3.9808 | 1.03572 | -0.869 | 0.107 | 0.192 | 0.214 |
| VAR00050 | 4.0019 | 1.05521 | -0.724 | 0.107 | -0.541 | 0.214 |
| VAR00051 | 4.0115 | 1.03583 | -0.784 | 0.107 | -0.235 | 0.214 |
| VAR00052 | 3.977 | 1.11089 | -0.825 | 0.107 | -0.291 | 0.214 |
| VAR00053 | 3.6948 | 1.25152 | -0.637 | 0.107 | -0.678 | 0.214 |
| VAR00054 | 3.7831 | 1.14564 | -0.6 | 0.107 | -0.592 | 0.214 |
| VAR00055 | 3.7562 | 1.13852 | -0.602 | 0.107 | -0.514 | 0.214 |
| VAR00056 | 3.8023 | 1.11168 | -0.573 | 0.107 | -0.56 | 0.214 |
| Valid N (listwise) |  |  |  |  |  |  |
